# A large-scale screening campaign of putative carbohydrate-active enzymes reveals a novel xylanase from anaerobic gut fungi

**DOI:** 10.1128/mbio.01007-25

**Published:** 2025-08-05

**Authors:** Shiyan Jin, Isabella R. Farrand, Yan Chen, Jennifer W. Gin, Bo Zhang, Elaine Kirschke, Christopher J. Petzold, Paul D. Adams, Michelle A. O'Malley

**Affiliations:** 1 Department of Chemical Engineering, University of California 523353, Santa Barbara, California, USA; 2 Biological Systems and Engineering Division, Lawrence Berkeley National Laboratory 1666, Berkeley, California, USA; 3 Joint BioEnergy Institute 124489, Emeryville, California, USA; 4 Molecular Biophysics and Integrated Bioimaging Division, Lawrence Berkeley Laboratory 1666 , Berkeley, California, USA; 5 Department of Bioengineering, University of California 224036, Berkeley, California, USA; 6 Department of Bioengineering, University of California 8786, Santa Barbara, California, USA; Max Planck Institute for Marine Microbiology, Bremen, Germany

**Keywords:** heterologous expression, screening, carbohydrate-active enzymes, anaerobic fungi, protein function prediction, enzyme characterization

## Abstract

The genomes of anaerobic gut fungi (AGF) encode a diverse array of carbohydrate-active enzymes (CAZymes), yet exceedingly few of these enzymes have been experimentally validated or expressed in heterologous systems. Here, we developed a predictive bioinformatic pipeline to annotate novel putative CAZymes from anaerobic fungi and validate their activity through large-scale heterologous expression in *Escherichia coli*. A total of 173 fungal proteins from *Piromyces finnis* associated with biomass degradation were synthesized and expressed in *E. coli*, and 9.8% were soluble with expression levels exceeding 5% of the total proteome using high-throughput proteomic screening. Among these 17 heterologously expressed proteins, analysis with AlphaFold and FoldSeek predicted 13 multi-functional proteins containing catalytic domains fused with repetitive fungal dockerins, and half of the substrate predictions were experimentally validated. One promising enzyme, celsome_012, exhibited robust and specific activity against beechwood xylan at 37°C and pH 6.4, with titers that were also fivefold higher than those of other recombinant proteins screened here. Both Michaelis-Menten kinetics and the linearized Lineweaver-Burk equation yielded consistent values for *K*_m_, and its activation energy was estimated at 51.9 kJ/mol based on the Arrhenius model. This work supports the industrial translation of anaerobic fungal CAZymes due to their robust lignocellulolytic activity and provides a framework for prioritizing AGF proteins for efficient *E. coli* heterologous expression.

**IMPORTANCE:**

Efficient breakdown of plant biomass is crucial for producing high-value bio-based products, but identifying enzymes that reduce deconstruction costs remains a challenge. In this study, we harnessed novel CAZymes encoded in the AGF genome through high-throughput proteomic screening for CAZyme expression to identify promising fungal enzymes suitable for large-scale production in *E. coli*. Additionally, we leveraged cutting-edge computational tools to predict enzyme structure and function, accelerating the screening process beyond traditional methods. Experimental validation confirmed the accuracy of these predictions and revealed a highly active novel xylanase, expanding the available enzyme toolbox for biomass conversion. Overall, this study represents a comprehensive large-scale screening campaign of putative AGF CAZymes, highlighting proteins amenable to *E. coli* overexpression, integrating advanced sequence and structural annotation, and identifying a robust, novel fungal xylanase for detailed biochemical characterization.

## INTRODUCTION

**Fig 1:**
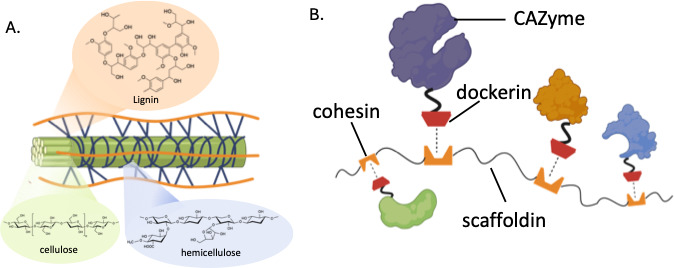
Lignocellulose is a complex biopolymer that is degraded in nature by carbohydrate-active enzymes and carbohydrate-active enzyme complexes (cellulosomes). (**A**) Lignocellulose is composed of carbohydrate polymers (cellulose and hemicellulose) and aromatic lignin deposits that provide structure and enzyme recalcitrants to plants. (**B**) An illustration of a cellulosome (multi-enzyme complex) structure whereby non-catalytic dockerin domains are fused to carbohydrate-active enzymes (CAZymes) that enable enzymes to associate with complementary cohesion domains on a flexible scaffold. Figure generated in BioRender.

Lignocellulose, which accounts for up to 50% of plants’ total dry weight, is the most available raw material for biofuel and bio-based chemical production (1–3). As the major component of plant cell walls, lignocellulosic biomass provides plants with rigidity, mechanical strength, and protection against parasites and other environmental factors (1, 4). Even though the chemical composition varies among plant species, it consists of three primary polymers: cellulose, hemicellulose, and lignin, as illustrated in Fig. 1A (5). Among the three, cellulose is the most abundant and has the simplest structure composed of a chain of glucose linked by β(1→4) bonds (1). Hemicellulose monomer, such as xylose and galactose, has a more diverse structure, depending on the plant type (6). The structural complexity of lignin, a heterogenous aromatic polymer, is responsible for the recalcitrance of lignocellulose and makes it exceptionally difficult to valorize (7–9).

Innovations in microbial bioprocessing have the potential to meet the rising demand for chemicals and fuels from sustainable sources (10). Besides the widely used model organisms deployed in industry, a wealth of undercharacterized microorganisms, such as anaerobic gut fungi (AGF) under phylum *Neocallimastigomycota*, are attractive alternatives as production systems and/or enzyme sources owing to their specialization in plant biomass deconstruction (10, 11). AGF are early-diverging lineages of fungi retaining distinct phenotypic traits (12, 13). In their native environment, these fungi exhibit a symbiotic relationship with large herbivores, living in the host’s gut or rumen and relying on the ingested lignocellulosic vegetation as substrates (10). In return, AGF assists in the digestion process to reduce the host’s burden by releasing simple carbohydrates and fermentation products from biomass (13–16).

Compared to commercially deployed fungal strains that require lignocellulose pretreatment, such as *Aspergillus niger* (*Agaricomycetes*) and *Trichoderma reesei* (*Sordariomycetes*), many enzymes secreted by the AGF (*Neocallimastigomycetes*) are more effective in raw biomass breakdown (up to 50%) without any pretreatment (12, 13, 17–22). Unique to anaerobic systems, AGF further leverage their rich reservoir of carbohydrate-active enzymes (CAZymes) by co-localizing most of them into large multi-protein complexes referred to as cellulosomes that function as potent lignocellulose-degrading machines, similar to schemes used by many anaerobic bacteria (17, 23). Cellulosomes usually contain one or more scaffoldin proteins with cohesin repeats that recruit different dockerin-fused CAZymes together through complementary cohesin-dockerin binding (Fig. 1B) (24). Many CAZymes are tethered by a fungal dockerin domain that binds to cohesin when recruited through the scaffoldin (25, 26) (Fig. 1B). Compared to the reaction catalyzed by freely diffusing enzymes, the associated CAZymes initiate lignocellulose degradation at an accelerated rate due to the proximity effect (23, 24), a promising potential framework for industrial biomass processing.

Despite the diverse array of CAZymes putatively encoded in the AGF genome for efficient lignocellulosic degradation (13), many CAZymes have evaded sequence-based annotation because their unique protein sequences lack reference information. Meanwhile, recent advances in *in silico* protein folding algorithms and annotation, such as AlphaFold and FoldSeek, enable function prediction based on structure homology rather than sequence homology alone (27, 28), particularly in identifying small and flexible regions, such as fungal dockerins. By combining both sequence and structure prediction, we can achieve a more comprehensive understanding of a protein’s putative function in non-model organisms such as AGF.

Studies have shown that anaerobic fungi respond dynamically to changes in their surrounding substrates by adapting cellulosome compositions (19, 23), elucidating the cruciality to unmask the function of fungal CAZymes and uncover their potential. Unfortunately, few AGF proteins have been expressed, purified, and kinetically characterized (29–31) due to the lack of genetic engineering tools compatible for the native host. Fortunately, heterologous protein expression in model chassis organisms is a well-established approach for protein characterization and mass production (32). As a preferred system for producing recombinant proteins, *Escherichia coli* is favored for its short doubling time, well-understood biology, and relatively straightforward cloning processes (32, 33). *E. coli* can accommodate not only prokaryotic proteins but also some complex ones from eukaryotic origins, such as human insulin (34). However, major challenges posed by *E. coli* include low expression levels of proteins with rare codon usage, the formation of insoluble protein aggregates due to misfolding at high expression levels, and loss of activity because of its inability to confer post-translational modification (35, 36).

In this study, we screened the expression of 173 putative CAZyme constructs identified from AGF in *E. coli* and evaluated their expression level through proteomic analysis to select for those suitable for *E. coli* production. Sequence and structure homology were then employed to predict functions for the panel of expressed proteins, with confirmation from activity assays. Altogether, this effort led to the validation of several AGF CAZymes, including the identification and kinetic characterization of a novel xylanase sourced from AGF. This work presents a promising step toward translating anaerobic fungal enzymes to industrial applications and waste valorization.

## MATERIALS AND METHODS

### Strains, plasmids, and reagents

A total of 173 putative CAZymes were synthesized and denoted as celsome_001 to celsome_173, which were collated from previous studies that identified transcriptionally upregulated and fungal dockerin-fused proteins from the anaerobic fungus *Piromyces finnis* (13, 16) (Supplemental material). cDNA fragments for putative AGF genes were synthesized into the pET-28a expression vector after codon optimization tailored for the *E. coli* system with the sequence for an N-terminal His-tag of MGSSHHHHHHSSGLVPRGSHMAS. Plasmids were transformed into *Escherichia coli* BL21(DE3) at the Dept. of Energy Joint Genome Institute (JGI), USA.

For substrate-based enzymatic activity assays, carboxymethyl cellulose (CMC) was purchased from Sigma-Aldrich (St. Louis, MO, USA); xyloglucan was purchased from Fisher Scientific (Waltham, MA, USA). Beechwood xylan was purchased from Megazyme (Bray, Ireland). Arabinogalactan was purchased from TCI (Tokyo, Japan).

### Initial protein expression screening in *E. coli*

*E. coli* BL21(DE3) strains overexpressing each recombinant putative CAZyme were grown in 1 mL of Luria-Bertani (LB) medium containing 50 µg/mL kanamycin for plasmid maintenance. After 1 h of growth in a rotary shaker (200 rpm) at 37°C, cells were induced for expression with 0.1 mM isopropyl β-D-1-thiogalactopyranoside (IPTG) for another 1 h in 96-well plates. Cells were pelleted and stored at −80°C until later proteomic analysis.

Protein was extracted from cell pellets, and tryptic peptides were prepared by following an established proteomic sample preparation protocol (37). Briefly, cell pellets were resuspended in Qiagen P2 Lysis Buffer (Qiagen, Germany) to promote cell lysis. Proteins were precipitated with the addition of 1 mM NaCl and 4× vol acetone, followed by two additional washes with 80% acetone in water. The recovered protein pellet was homogenized by pipetting mixing with 100 mM ammonium bicarbonate in 20% methanol. Protein concentration was determined by the DC protein assay (Bio-Rad, USA). Protein reduction was accomplished using 5 mM Tris 2-(carboxyethyl)phosphine for 30 min at room temperature, and alkylation was performed with 10 mM iodoacetamide (final concentration) for 30 min at room temperature in the dark. Overnight digestion with trypsin was accomplished with a 1:50 trypsin:total protein mass ratio (wt/wt). The resulting peptide samples were analyzed on an Agilent 1290 UHPLC system coupled to a Thermo Scientific Orbitrap Exploris 480 mass spectrometer for discovery proteomics (38). Briefly, peptide samples were loaded onto an Ascentis ES-C18 Column (Sigma-Aldrich) and were eluted from the column by using a 10 min gradient from 98% solvent A (0.1% vol/vol formic acid) and 2% solvent B (0.1% formic acid in acetonitrile) to 65% solvent A and 35% solvent B. Eluting peptides were introduced to the mass spectrometer operating in positive-ion mode and were measured in data-independent acquisition (DIA) mode with a duty cycle of three survey scans from *m*/*z* of 380–985 and 45 tandem mass spectrometry (MS2) scans with precursor isolation width of 13.5 *m*/*z* to cover the mass range. DIA raw data files were analyzed by an integrated software suite DIA-NN (39). The database used in the DIA-NN search (library-free mode) was *E. coli* latest Uniprot proteome FASTA sequences plus the protein sequences of the heterologous proteins and common proteomic contaminants. DIA-NN determines mass tolerances automatically based on first pass analysis of the samples with automated determination of optimal mass accuracies. The retention time extraction window was determined individually for all MS runs analyzed via the automated optimization procedure implemented in DIA-NN. Protein inference was enabled, and the quantification strategy was set to robust LC = high accuracy. Output main DIA-NN reports were filtered with a global false discovery rate (FDR) set at 0.01 (FDR ≤0.01) on both the precursor level and protein group level. The top 3 method, which is the average MS signal response of the three most intense tryptic peptides of each identified protein, was used to plot the quantity of the targeted proteins in the samples (40). We monitored the production of targeted protein to analyze the CAZyme expression in *E. coli*. Protein constructs where the target protein exceeded 5% of the total proteome were considered highly expressed, and those less than 5% were considered poorly expressed. We also measured the production of small heat-shock protein A, IbpA, alongside the targeted proteins, as an indicator of cell stress level. Protein constructs were considered insoluble if the associated IbpA production constituted more than 0.5% of the total proteome, a threshold determined empirically.

### Dinitrosalicylic acid enzymatic activity screening for recombinant CAZyme constructs

The well-expressed CAZymes in *E. coli* were screened for their cellulase and hemicellulase activities. They were heterologously expressed in 5 mL LB medium after 0.1 mM IPTG induction at 37°C for 1 h, reaching an optical density of 2.0 ± 0.5 per mL. Cell lysates, prepared by sonication, were used in conducting reactions with model cellulose (CMC) and hemicellulose (beechwood xylan and xyloglucan). Product concentration, measured as the reducing sugar in the reaction mixture, was examined using the dinitrosalicylic acid (DNS) assay (41). For activity screening of the 17 selected constructs on CMC, beechwood xylan, and xyloglucan, 175 µL reactions in 0.1 M phosphate-buffered saline (PBS), pH 7.2, containing 100 µL of cell lysate and 75 µL 2% wt/vol substrate were statically incubated at 39°C for 24 h. Briefly, after adding 50 µL of DNS to a 150 µL reaction sample and incubating at 95°C for 5 min, the mixture was measured at 540 nm absorbance using a Tecan Infinite M1000 plate reader (Tecan Group, Männedorf, Switzerland).

### Enzyme cloning, expression, and purification for kinetic characterization

One liter of *E. coli* BL21(DE3) cells overexpressing the construct celsome_012 was induced with 0.1 mM isopropyl β-D-1-thiogalactopyranoside at 37°C for 1 h. Following induction, cells were lysed using Sonifier SFX250 (Branson) with Halt Protease Inhibitor Cocktail (Thermo Scientific) for 10 min at 30% amplitude. Unlysed cells were removed by centrifugation, and the cell lysate was filtered through a 0.2 µm filter before loading onto a nickel-nitrilotriacetic acid HisTrap column (Cytiva Life Sciences, Marlborough, MA, USA) equilibrated with PBS buffer (pH 7.5) with 20 mM imidazole. The His-tagged proteins were then eluted with 300 mM imidazole in PBS before buffer change into 50 mM Tris buffer, pH 7.2. The eluted fraction containing xylanase activity was collected and confirmed using sodium dodecyl sulfate polyacrylamide gel electrophoresis using 4%–15% Mini-PROTEAN TGX Precast Protein Gels (Bio-Rad) under a 150 V current for 1 h, and then the protein bands were stained with Coomassie Brilliant Blue (42).

### Protein function prediction by sequence homology and post-translational modification prediction *in silico*

CAZyme substrates were predicted from protein sequences using the dbCAN3-sub package (43). Protein functions were predicted using the HMMER model and DIAMOND model incorporated in the dbCAN3 package. N-linked glycosylation sites were predicted using MusiteDeep (44–46), and phosphorylation sites were predicted using NetPhos v.3.1 (47).

### *In silico* protein folding and function prediction by structure homology

Protein structures were predicted using AlphaFold (version 2.2) using a NVIDIA A100 GPU with 80 GB of memory. For each target sequence, five predictions were generated using default parameters (27). The highest-ranked prediction for each target was selected for further analysis. The predictions were processed to remove low-confidence regions and segmented into likely domains using the phenix.process_predicted_model program (48) from Phenix software (49). Default parameters were used for the removal of low-confidence regions. For domain identification, the following parameters were used: pae_cutoff = 4, pae_graph_resolution = 4, pae_power = 2, single_letter_chain_ids = True, minimum_domain_length = 20, minimum_sequential_residues = 10. The resulting domains were then each analyzed using the FoldSeek program (command line v.2ad017897d3dab66dd33ea675e92215bdfb4a64d) (28). The easy-search option was used with the FoldSeek provided Protein Data Bank (50) database (pdb100, v.2023-03-20) with --alignment-type 1 and the following output parameters: target, fident, bits, evalue, prob, qstart, qend, qlen, tstart, tend, tlen. Where Foldseek suggested related experimental structures for a domain, the Protein Data Bank ID was used in a REST query to the Research Collaboratory for Structural Bioinformatics PDB to retrieve the following information: text description of the structure, functional annotation, enzyme class, source organism, and oligomeric state. The annotation used InterPro or PFam fields, depending on which was available. This information was combined with the FoldSeek results to generate a ranked list of potential structurally related proteins for each domain of each target, ranked by the FoldSeek *e* value. The results were visually inspected to determine potential functions for each target and were compared to the sequence-based annotation. The process of analyzing the predicted structures was facilitated by the creation of a series of Python and C-shell scripts.

### Determining kinetic properties of CAZymes

The optimal temperature of celsome_012 was measured via a 3 h incubation of 1.5 ng/µL of purified celsome_012 proteins with 2% wt/vol beechwood xylan in 50 mM 2-morpholinoethanesulfonic acid (MES) buffer (pH 6.5) across a temperature range of 4°C–55°C. The most ideal pH was determined via a 2 h incubation at 39°C of the same protein and substrate concentration in 50 mM of different buffers. The buffer usages were sodium citrate (pH 3.0–6.0), MES (pH 6.5), Tricine (pH 7.0–9.0), and sodium bicarbonate (pH 10.5). As products had accumulated to levels sufficient for reliable quantification by 2 and 3 h, the selection of time points was based on experimental convenience.

The substrate specificity of celsome_012 was examined by incubating with 1.5 ng/µL of purified celsome_012 proteins with 2% wt/vol substrates in 50 mM MES buffer (pH 6.5) at 39°C for 24 h. Substrates included in this study were CMC, phosphoric acid swollen cellulose, beechwood xylan, and mixed-linkage (1,3;1,4)-β-d-glucan (MLG, also known as β-glucan). The kinetic parameters of celsome_012 were calculated by monitoring the product formation over 8 h at different saturated beechwood xylan concentrations from 1,000 to 2,500 µM.

### Bioinformatic analysis of putative xylanase

The molecular weights of the recombinant proteins were predicted at Bioinformatics.org (https://www.bioinformatics.org/sms2/protein_mw.html). Proteins closely related to protein celsome_012 sequences in the databases for the candidate cellulases were identified with BlastP UniProt. Module structures of the celsome_012 were predicted by AlphaFold (27). Amino acids of celsome_012 and their highest matches from the Blast analyses were aligned for phylogenetic analyses. The phylogenetic tree was generated with UniProt and visualized using iTOL v.6 (https://itol.embl.de/).

## RESULTS

### Assessing the heterologous expression efficiency of fungal CAZymes in *E. coli*

**Fig 2:**
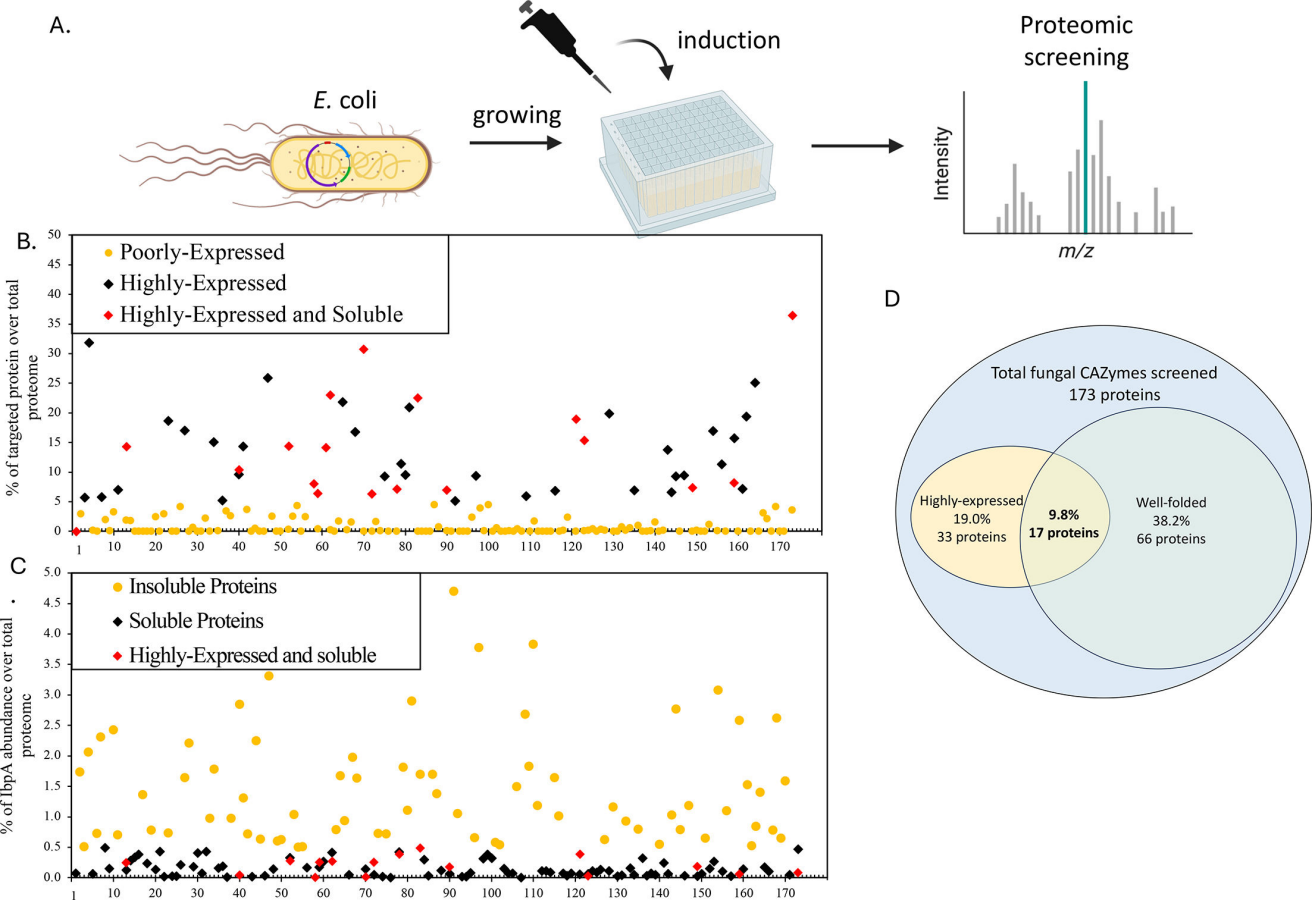
A small fraction of recombinant AGF proteins were solubly expressed at significant levels in *E. coli* as assessed using untargeted proteomics on cell lysates. (**A**) Workflow for proteomic screening of 173 CAZymes identified from AGF strain *P. finnis*. (**B**) Distribution of fungal CAZyme expression levels in *E. coli*. Proteins with over 5% of total proteome were considered highly expressed, while those with less than 5% were considered poorly expressed. (**C**) Distribution of IbpA production during fungal protein expression in *E. coli* was used as an indicator of protein folding success and solubility. IbpA levels below 0.5% of the total proteome indicated soluble heterologous protein production, while levels above 0.5% indicated insoluble/aggregated heterologous proteins. (**D**) Seventeen highly and soluble proteins from AGF were further characterized in *E. coli*. Panel A was made in BioRender.

A total of 173 putative fungal CAZymes genes from *Piromyces finnis*, previously identified for being associated with lignocellulose degradation (13), were heterologously expressed in *E. coli*. The relative abundance and folding quality of these fungal proteins were assessed using proteomics analysis (Fig. 2A). Among the 173 CAZymes, only 28.8% of the proteins were categorized as highly expressed in the *E. coli* system (Fig. 2B; Fig. S1A). Soluble protein folding was further evaluated by monitoring the expression level of IbpA, whose presence indicates high cellular stress that is often associated with the formation of insoluble inclusion bodies (51, 52). Of the screened CAZymes, 48% were expressed without inducing significant stress on the host cells, and therefore, these were considered soluble proteins (Fig. 2C; Fig. S1B). Considering both expression level and solubility, only 9.8% of the fungal proteins were deemed suitable for heterologous expression in *E. coli* (Fig. 2D). This selection process yielded a subset of 17 well-expressed and soluble protein candidates for further analysis (Table 1).

**TABLE 1:** List of predicted protein functions from sequence annotation and structure annotation^*a*^

Gene ID	Uniprot ID	Sequence function prediction	Structure function prediction	Catalytic domain EC
celsome_012	A0A1Y1VE81	GH8	GH8, CBM10, CBM10	3.2.1.156
celsome_039	A0A1Y1UV42	CE1, CBM13	CE1, CBM13, CBM10	3.2.1.73
celsome_051	A0A1Y1UYQ1	CBM13	Peptidase S9, CBM13	3.1.1.1
celsome_057	A0A1Y1VER0	GH5_1	GH5, CBM10	3.2.1.4
celsome_058	A0A1Y1UXF1	GH9, fungi_dockerin	GH9, CBM10, CBM10, CBM10	3.2.1.4
celsome_060	A0A1Y1V856	GH6, fungi_dockerin	GH6, CBM10, CBM10	3.2.1
celsome_061	A0A1Y1VLQ1	GH10, CBM13, fungi_dockerin	GH10, CBM13, CBM10, CBM10	3.2.1.8
celsome_069	A0A1Y1VL05	GH10, CBM13	GH10, CBM13, CBM10, CBM10	3.2.1.8
celsome_071	A0A1Y1VKI6	GH6	GH6, GH6, CBM10, CBM10	3.2.1
celsome_077	A0A1Y1VJR3	GH9	GH9, CBM10, CBM10	3.2.1.4
celsome_082	A0A1Y1VM89	fungi_dockerin, fungi_dockerin	Leucine-rich repeat, CBM10, CBM10	2.7.11.1
celsome_089	A0A1Y1VQ34	GH95, CBM13	GH95, CBM13	3.2.1.63
celsome_120	A0A1Y1UXM1	fungi_dockerin	CBM10, CBM10	na^*b*^
celsome_122	A0A1Y1V4G5	GH5_4	GH5, CBM10	3.2.1.4
celsome_148	A0A1Y1U × 92	CE1	CE1	3.1.1.73
celsome_158	A0A1Y1VQ18	GH5_1, fungi_dockerin	GH5, CBM10, CBM10, CBM10	3.2.1.4
celsome_172	A0A1Y1VK22	CE6, CBM22, fungi_dockerin, fungi_dockerin	CE6, CBM22, CBM10, CBM10	3.2.1.8

^*a*^ CBM10 is considered a dockerin in fungi.

^*b*^ na indicates not applicable.

Further investigation into the properties of the selected 17 proteins provided valuable insights for developing a pipeline to identify fungal proteins amenable to bacterial expression. Comparative analysis of these expressed proteins against the full heterologous protein population revealed no significant differences in molecular weight or length distribution (Fig. S2A and S2B). Additionally, predicted post-translational modifications by MusiteDeep and NetPhos v.3.1 indicated that the number of N-linked glycosylation and phosphorylation sites did not predetermine the outcome of heterologous expression (Fig. S2C and D).

### Protein function prediction using sequence and structure homology annotation

**Fig 3:**
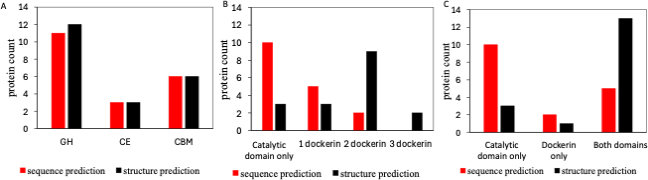
Integrating both dbCAN3 and AlphaFold/FoldSeek achieves a more comprehensive prediction of protein function in 17 AGF candidate CAZymes expressed in *E. coli*. (**A**)Total number of non-dockerin domains predicted by sequence and structure prediction pipeline. CBM, carbohydrate-binding module; CE, carbohydrate esterase; GH, glycoside hydrolase. (**B**) Number of dockerin domains predicted per protein by sequence and structure prediction pipeline. (**C**) Number of proteins with different domains identified by sequence prediction and structure prediction pipelines.

Functions of the selected 17 AGF proteins prioritized for their high expression and solubility in *E. coli* were predicted using the hidden Markov model from package dbCAN3 based on sequence homology against other known proteins. A total of 11 glycoside hydrolase (GH) domains were identified, more than the number of carbohydrate esterase (CE) domains and carbohydrate-binding module (CBM) domains, suggesting hydrolysis is the key step, aided by substrate targeting and ester bond cleaving (Fig. 3A; Table 1). Besides the hydrolytic domains, seven CAZymes were found to possess fungal dockerin domains, suggesting association in fungal cellulosomes.

AlphaFold and FoldSeek were employed in combination with sequence annotation to achieve a more comprehensive prediction of protein function. For catalytic domains, structural predictions majorly aligned with sequence-based annotation in predicting GH, CE, and CBM domains (Fig. 3A, Table 1). Compared to sequence homology analysis that identified 10 CAZymes without a dockerin domain, structural prediction decreased the number to 3 CAZymes without dockerin, with 11 CAZymes containing repetitive dockerins (Fig. 3B). Additionally, structural homology analysis identified 13 proteins with both domains, more than twice the number of the five CAZymes with both domains found in the sequence analysis (Fig. 3C). Therefore, integrating structure-based prediction along with more traditional sequence-based prediction enabled a more complete prediction of the AGF CAZyme function.

### Experimental validation of predicted substrate targets

**Fig 4:**
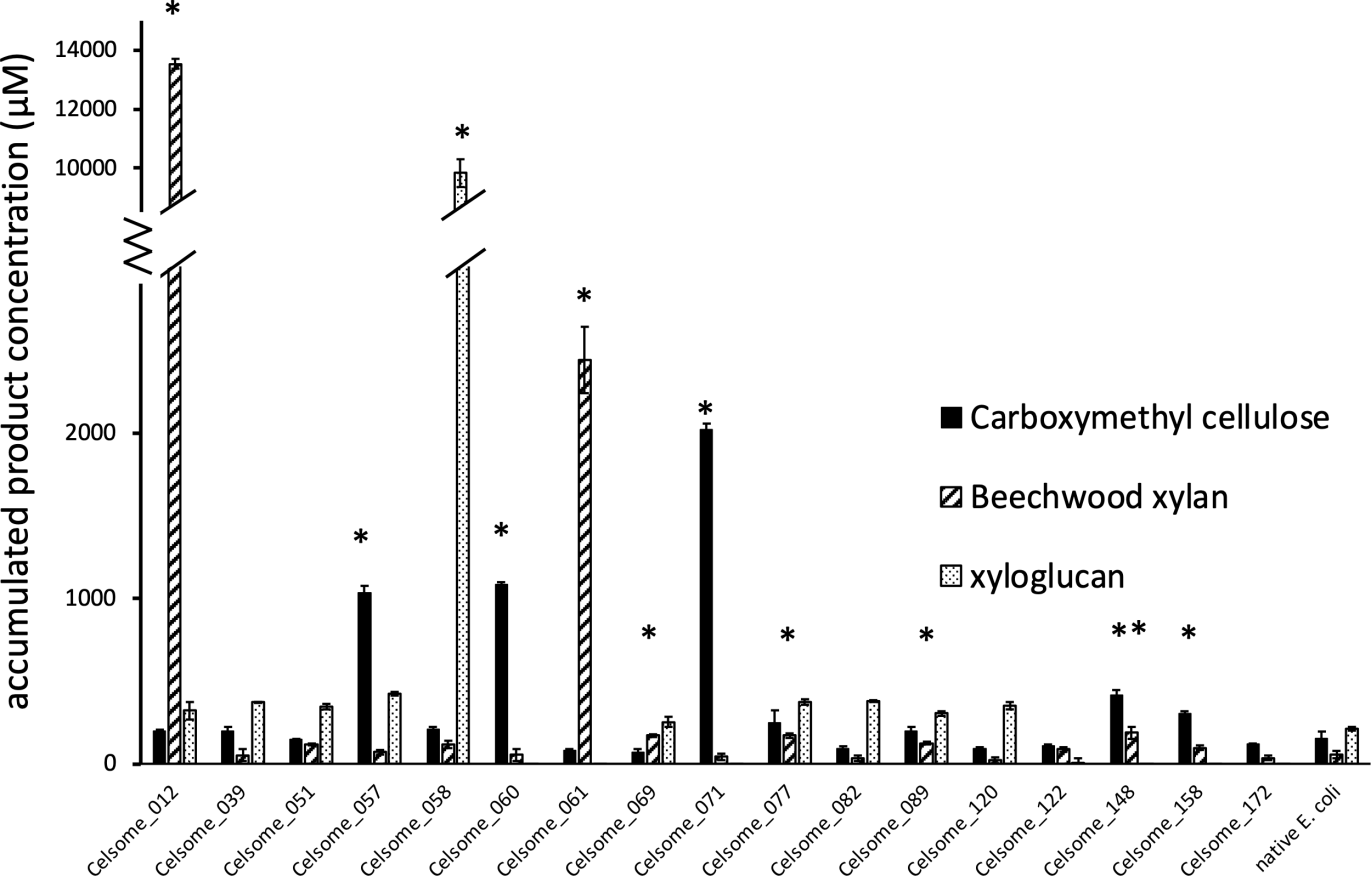
Experimental validation of heterologously expressed AGF cellulase and hemicellulases activity using (DNS) substrate screening assay against model cellulose (carboxymethyl cellulose), model hemicellulose (beechwood xylan), and a combination of both (xylogalactan). *, the final accumulated product is higher than native *E. coli* with at least 90% confident level. **, the accumulated product is above the baseline (negative control) at *P* < 0.1.

The *in silico* substrate prediction for each AGF protein made by dbCAN3 was subsequently validated through hydrolytic activity assay against predicted substrates. Ten out of 17 proteins screened demonstrated activity against one or more substrates, as indicated by an increased release of reducing sugars compared to native *E. coli* BL21(DE3) lysates in a DNS assay (Fig. 4). The activity screening identified 4 cellulases, 6 xylanases, and 1 xyloglucanase, validating 6 out of the 14 dbCAN3 predictions and highlighting the potential for enhancements in annotation algorithms for anaerobic fungal CAZymes (Table 2). Notably, a multi-functional protein, celsome_148, demonstrated activity against both cellulose and hemicellulose, despite dbCAN3 failing to predict these targeted substrates. Additionally, one particularly robust xylanase, celsome_012, produces five times more product than celsome_061, the second-most active xylanase screened in this study.

**TABLE 2:** List of predicted targeting substrates for CAZymes and their associated experimental validation

Gene ID	Predicted substrate by dbCAN	Experimental validated substrates	Agreement with prediction or not
celsome_012	Xylan, β-glucan	Xylan	Agree
celsome_039	Xylan	–^*a*^	–
celsome_051	Xylan	–	–
celsome_057	β-Glucan	Xylan	Disagree
celsome_058	Cellulose	Xylogalactan	Disagree
celsome_060	Cellulose	Cellulose	Agree
celsome_061	Xylan	Xylan	Agree
celsome_069	Xylan	Xylan	Agree
celsome_071	Cellulose	Cellulose	Agree
celsome_077	Cellulose	Xylan	Disagree
celsome_082	Xylan	Cellulose	Disagree
celsome_089	Pectin, xyloglucan, xylan	Xylan	Agree
celsome_120	–	–	–
celsome_122	–	–	–
celsome_148	–	Cellulose, xylan	New
celsome_158	β-Glucan	Cellulose	Disagree
celsome_172	Xylan, β-glucan	–	–

^*a*^ –, not applicable.

### Biological and kinetic characterization of xylanase celsome_012

**Fig 5:**
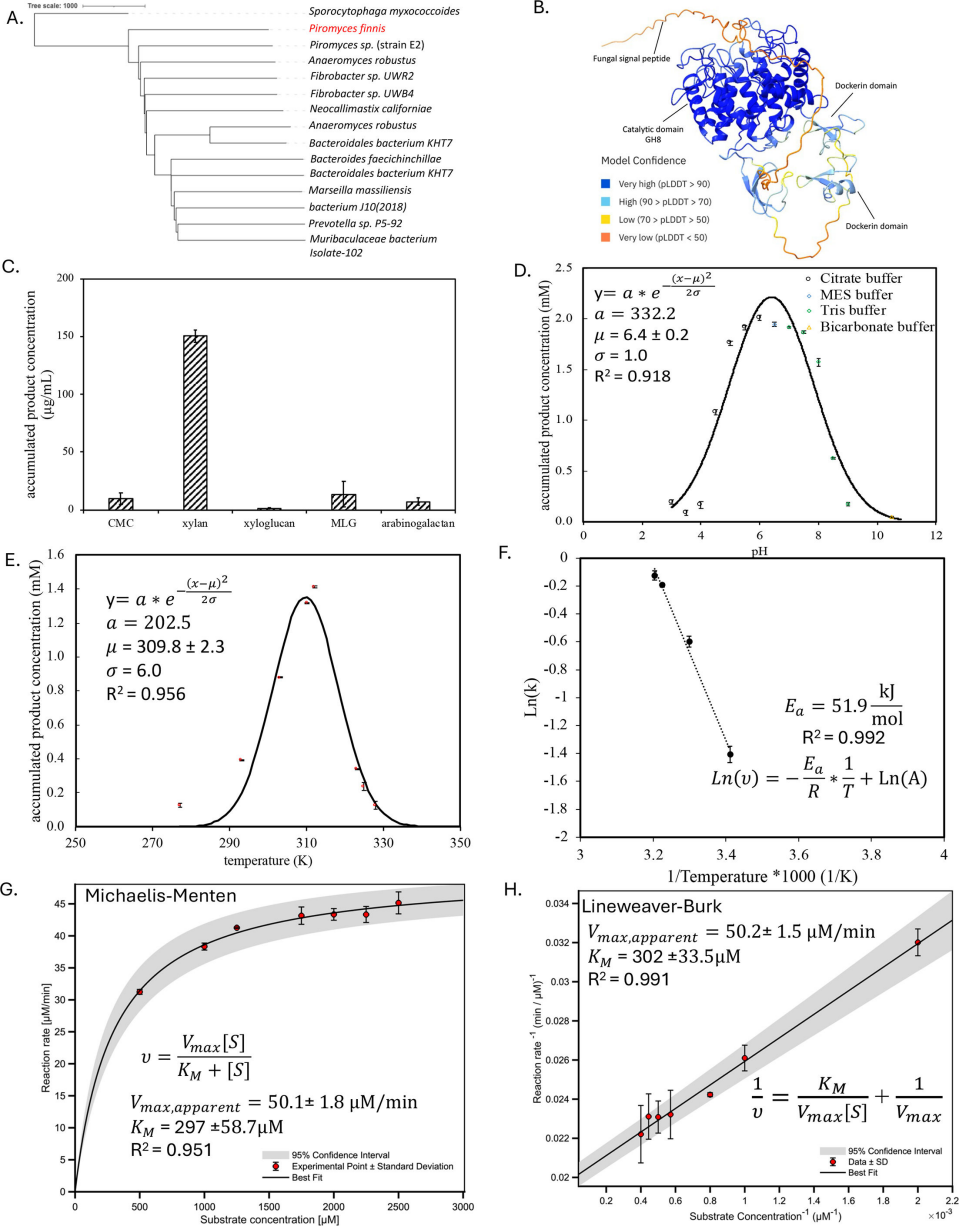
Biological and kinetic characterization of critical functional parameters of AGF protein celsome_012. (A) A phylogenetic tree depicting the evolutionary relationship of protein celsome_012 (Uniprot ID: A0A1Y1VE81). (**B**) A predicted protein structure generated using AlphaFold 2.2. (**C**) Substrate specificity analysis of celsome_012 revealing highest enzyme activity against beechwood xylan in comparison to additional substrates screened, including carboxymethyl cellulose (CMC), xyloglucan, mixed-linkage (1,3;1,4)-β-d-glucan (MLG), and arabinogalactan. (**D**) Gaussian model predicting the activity profile of celsome_012 over a pH range from 3.0 to 10.0. The optimal pH is reported with a range of 95% confidence interval (CI). (**E**) Gaussian model predicting the activity profile of celsome_012 over a temperature range from 277 to 328 K. The optimal temperature is reported with a range of 95% CI. (**F**) Calculation of the activation energy for celsome_012 using the Arrhenius model. (**G**) Determination of key enzyme kinetic parameters through modeling with the Michaelis-Menten equation. (**H**) Determination of key enzyme kinetic properties using the linearized Lineweaver-Burk equation. The error bars represent the standard deviation from triplicates.

Among the 17 constructs screened, celsome_012, an experimentally confirmed xylanase (Fig. 4), accumulated more than 13 mM product within 24 h, significantly more than any other construct screened here (Fig. 4). The phylogenetic tree of celsome_012 suggests it is conserved in four AGF genera from the *Neocallimastigomycetes* phylum (*Piromyces finnis*, *Piromyces* sp., *Anaeromuces robustus*, and *Neocallimastix californiae*) while diverging from other bacterial xylanases (Fig. 5A) (12). AlphaFold-predicted structure of celsome_012 suggests a core globular hydrolytic domain, followed by two repetitive fungal dockerin domains tethered at the C-terminus (Fig. 5B). A long linker at the N-terminal is partially predicted as a native fungal signal peptide by SignalP (Fig. 5B). BLAST analysis against the UniProt protein database reveals that this xylanase shares minimal sequence similarity with bacterial proteins, and the highest homology of the entire protein is 72.7% alignment identity with a glycoside hydrolase from *Fibrobacter* sp. UWB4 (GenBank no. WP_088639271.1).

Different substrates were screened against celsome_012, and the result suggests exclusive targeting on xylan (Fig. 5C). The optimal reaction conditions were determined by assaying a range of temperatures (4°C–55°C) and pH values (pH 3.0–10.5). The optimal temperature and pH were found to be 37°C ± 2.3°C and pH 6.4 ± 0.2 modeled using a Gaussian model at a 95% confidence interval (Fig. 5D and E). Within the range of 30°C–39°C, the Arrhenius model was used to calculate the activation energy (*E*_A_) to be 51.9 kJ/mol (Fig. 5F).

Celsome_012 was incubated with saturated beechwood xylan, and the reaction rates were measured and later modeled using the Michaelis-Menten model. This analysis yields an apparent maximum velocity (*V*_max_) of 50.1 ± 1.8 µM/min and a Michaelis-Menten constant (*K*_m_) of 297.0 ± 58.7 µM (Fig. 5G). The Lineweaver-Burk double reciprocal model, applied to the same data set, returns similar *K*_m_ (302.0 ± 33.6 µM) and apparent *V*_max_ (50.2 ± 1.5 µM/min) (Fig. 5H).

## DISCUSSION

To investigate the function of many putative CAZymes encoded in AGF genomes, 173 protein enzyme candidates were selected for heterologous expression in the bacterial host *E. coli*, chosen for its rapid growth and ease of use (32). These proteins were prioritized based on multi-“omics” analysis (13), as novel CAZymes could be overlooked using standard annotation pipelines. However, as a prokaryotic organism, *E. coli* lacks the machinery required for processing complex eukaryotic proteins (32). Proteomic screening was employed to assess the compatibility between the heterologous host and the selected proteins. From this analysis, 50 proteins surpassed 5% of the total *E. coli* proteome, half of the 10% benchmark for evaluating the protein expression level to increase the CAZyme candidate pool (53). Nonetheless, the structural complexity of fungal proteins can impose significant cellular stress on the host, often resulting in protein misfolding. Notably, expression levels of IbpA, a stress marker (51, 52), exceeded 0.5% of the total proteome, 42-fold higher than the baseline for native *E. coli*, suggesting that 52% of the selected fungal CAZymes may not achieve proper folding. Taking these factors into consideration, only 17 fungal CAZymes were successfully expressed in the current host. Even though there was inherent bias introduced by applying these cutoff thresholds, this step prioritized the selection of soluble proteins and narrowed down the list of candidates for further studies.

The functions of the 17 selected proteins were predicted using both conventional sequence homology analysis (via the dbCAN3 HMMer pipeline) and newly developed structural homology methods (e.g., AlphaFold and FoldSeek). Analogous to the whole-genome annotation of anaerobic fungi (16), our pipeline also revealed proteins encoding diverse types of lignocellulolytic enzymes, such as GH5 and CE1, suggesting that these fungi decompose biomass holistically by targeting multiple chemical bonds through a wide distribution of biochemical mechanisms. More GH domains were predicted than CE and CBM combined, suggesting hydrolysis is a key step in lignocellulose degradation. Anaerobic fungi are known to form cellulosomes, held together by non-covalent interaction between the cohesin domain on the scaffoldin and the dockerin domain on the CAZyme. Seventeen CAZymes were anticipated to contain at least one dockerin domain to form cellulosomes. However, dbCAN3 failed to predict any fungal dockerin on 10 out of the 17 proteins screened. This likely stems from the less extensive studies on binding modules compared to catalytic CAZyme domains, resulting in a smaller reference data set for *in silico* predictions, an inherent bias frequently encountered in computational annotations. On the other hand, structural annotations more accurately identified repetitive dockerin domains, overcoming challenges posed by the small size and highly repetitive nature of dockerin modules. Thus, a more comprehensive prediction of protein functions could be achieved by integrating sequence and structural annotation, each specialized in identifying catalytic or dockerin domain.

The hydrolytic activities of the 17 selected AGF proteins were screened against four substrates representing model cellulose (carboxymethyl cellulose and β-glucan), hemicellulose (beechwood xylan), and a mix of both (xyloglucan). Only 43% of the substrate predictions made by dbCAN3 were validated experimentally. The loss of activity may result from decreased enzyme activity when expressed heterologously, while the discrepancies between the predictions and observed activities likely reflect limitations in the predictive accuracy of dbCAN3 algorithms. Additionally, the high activity of celsome_012 positions this fungal xylanase as a promising candidate for efficient heterologous expression in bacterial platforms.

Purified celsome_012 was incubated with beechwood xylan at a concentration twice the calculated *K*_m_ value to ensure the velocities were measured under substrate-saturating conditions, allowing reactions to reach maximum velocity. The Michaelis-Menten equation was used to calculate apparent *V*_max_ and *K*_m_, yielding an *R*^2^ value of 0.951. Given the intrinsic uncertainty in the non-linear regression model, the Lineweaver-Burk linear regression returned similar kinetic values with an *R*^2^ value of 0.991, supporting a strong model fit. Compared to previously reported bacterial xylanases, the specific activity of celsome_012 is 33.5 U/mg (Table 3), which is lower than many reported values but remains within the range, suggesting the presence of residual impurities likely derived from host cells. Negative controls were included throughout the experiment to confirm that the observed activity originates from celsome_012 rather than background host activity. The *K*_m_ value falls within the range of reported values for other xylanases, reflecting a moderate substrate affinity. The apparent *V*_max_ is lower than that of many purified bacterial xylanases, indicating potential for further activity optimization. Meanwhile, given the impurities, the actual *V*_max_ is likely underestimated. It is worth noting that none of the host organisms of the listed xylanases in Table 3 are reported to produce cellulosomes, suggesting different lignocellulose degradation machineries employed compared to AGF and explaining the discrepancies in *K*_m_ between celsome_012 and the reported xylanases.

**TABLE 3:** Xylanase activity characterized in other studies^*a*^

Original host	Expression host	Substrates	Specific activity (U/mg)	*K*_m_ (mg/mL)	*V*_max_ (μmol/min/mg)	Reference
*Piromyces finnis*	*Escherichia coli* BL21 (DE3)	Beechwood xylan	33.5	5.94	na	This study
*Paenibacillus campinasensis* BL11	*Paenibacillus campinasensis* BL11	Oat spelt xylan	2,392.0	6.78	4,953	(54)
*Paenibacillus campinasensis* G1-1	*Paenibacillus campinasensis* G1-1	Birch wood xylan	1,865.50	5.86	1,696.9	(55)
*Paenibacillus barengoltzii*	*Escherichia coli* BL21 (DE3)	Beechwood xylan	410.2	2.04	402.9	(56)
*Paenibacillus barengoltzii*	*Escherichia coli* BL21 (DE3)	Birch wood xylan	294.6	2.19	292.8	(56)
*Aspergillus ficuum* AF-98	*Aspergillus ficuum* AF-98	Beechwood xylan	288.7	3.267	18.38	(57)
*Paenibacillus barengoltzii*	*Escherichia coli* BL21 (DE3)	Oat spelt xylan	286.2	2.51	376.1	(56)
*Paenibacillus barengoltzii*	*Escherichia coli* BL21 (DE3)	Beech xylan	105.0	0.6	na	(58)
*Paenibacillus barengoltzii*	*Escherichia coli* BL21 (DE3)	Insoluble corncob xylan	67.2	na	na	(56)
*Anoxybacillus kamchatkensis* NASTPD13	*Anoxybacillus kamchatkensis* NASTPD13	Beechwood xylan	33.0	0.7	66.64	(59)
*Aspergillus ficuum* AF-98	*Aspergillus ficuum* AF-98	Birch wood xylan	na	3.747	11.1	(57)
*Aspergillus fischeri* Fxn1	*Aspergillus fischeri* Fxn1	Birch wood xylan	na	4.88	588.0	(60)
*Paenibacillus campinasensis* G1-1	*Paenibacillus campinasensis* G1-1	Oat spelt xylan	na	9.07	941.15	(55)

^*a*^ na indicates not applicable.

Celsome_012 has high substrate specificity toward beechwood xylan compared to model cellulose (CMC and MLG), hemicellulose (arabinogalactan), and a mixture of both (xyloglucan). Beechwood xylan was chosen as model xylan based on its less-branched structure compared to oat spelt xylan and higher solubility compared to birchwood xylan. The protein stability test suggested that celsome_012 remained stable between pH 5.0 and 8.0, with the highest activity observed at 6.4, aligned with the horse gut pH, where *Piromyces finnis* was originally isolated from (61). The optimal temperature for celsome_012 was modeled to be 37°C, which is the growth temperature of *E. coli* and 2°C lower than the native growth temperature of AGF (16, 62). Within the temperature range of 303–312 K, celsome_012 demonstrated thermally activated behavior when substrate concentration is saturated. The *E*_A_, calculated using linear regression, was 51.9 kJ/mol, comparable to other xylanases (63).

### Conclusion

Anaerobic fungi are specialists in crude lignocellulosic biomass degradation, making their secreted CAZyms promising and cost-effective candidates for the lignocellulose valorization industry. From past transcriptomic analyses (13), we identified 173 putative anaerobic fungal CAZymes in *Piromyces finnis* for further characterization by heterologous expression in *E. coli* (13, 16). Despite *E. coli* incompatibility with most of the fungal protein expression candidates, a combination of *in silico* sequence and structure annotations provides comprehensive predictions of catalytic or fungal dockerin domains.

Among the 17 AGF protein constructs successfully expressed by *E. coli* heterologously, celsome_012 had an outstanding and exclusive activity against the xylan substrate. It is modeled to be the most active around 37°C and pH 6.4, with high specific activity and consistent *K*_m_ and apparent *V*_max_ values. Further enhancement of activity could be achieved through engineering the active site using directed evolution or switching to a eukaryotic expression system.

### Key points

- Anaerobic fungi encode a rich collection of novel lignocellulolytic enzymes.
- 9.8% of 173 fungal CAZymes were successfully expressed in *E. coli.*
- Structural homology-based prediction aids enzyme identification from AGF.
- The novel, highly active fungal xylanase celsome_012 was characterized.

## Data Availability

All relevant data generated and analyzed in this study are included in this article or the supplemental material. Plasmids encoding the enzyme constructs studied in this work are available upon reasonable request. The generated mass spectrometry proteomics data have been deposited to the ProteomeXchange Consortium via the PRIDE partner repository with the dataset identifier PXD061558 (64). DIA-NN is freely available for download from https://github.com/vdemichev/DiaNN.

## SUPPLEMENTAL MATERIAL

File S1 mbio.01007-25-s0001.xlsx Protein sequences of 173 synthesized cDNA constructs from AGF.

Supplemental material mbio.01007-25-s0002.docx Supplemental figures and tables.

ASM does not own the copyrights to Supplemental Material that may be linked to, or accessed through, an article. The authors have granted ASM a non-exclusive, world-wide license to publish the Supplemental Material files. Please contact the corresponding author directly for reuse.
