# Supplementary material for: A large-scale screening campaign of putative carbohydrate-active enzymes reveals a novel xylanase from anaerobic gut fungi
Source: mBio. 2025 Aug 5;16(9):e01007-25. doi: 10.1128/mbio.01007-25 (PMC12421816; doi:10.1128/mbio.01007-25)
Supplement: Supplemental material — Supplemental figures and tables. [file mbio.01007-25-s0002.docx]

**Supplementary Information: A large-scale screening campaign of putative carbohydrate-active enzymes reveals a novel xylanase from anaerobic gut fungi**

Shiyan Jin^a^, Isabella R. Farrand^a^, Yan Chen^b^, Jennifer W. Gin^b^, Bo Zhang^a^, Elaine Kirschke^a^, Christopher J. Petzold^b^, Paul D. Adams^c,d,e^, Michelle A. O’Malley^a,c,f^#

^a^ Department of Chemical Engineering, University of California, Santa Barbara, CA, 93106 USA

^b^ Biological Systems and Engineering Division, Lawrence Berkeley National Laboratory, Berkeley, CA, 94720, USA

^c^ Joint BioEnergy Institute, Emeryville, CA, 94608, USA

^d^ Molecular Biophysics and Integrated Bioimaging Division, Lawrence Berkeley Laboratory, Berkeley, CA, 94720 USA.

^e^ Department of Bioengineering, University of California, Berkeley, CA, 94720 USA

^f^ Department of Bioengineering, University of California, Santa Barbara, CA, 93106 USA

Running Head: Screening and Characterization of Novel Fungal CAZymes

# Address correspondence to Michelle A. O’Malley: [momalley@ucsb.edu](mailto:momalley@ucsb.edu\)

**SUPPLEMENTAL MATERIAL**

**
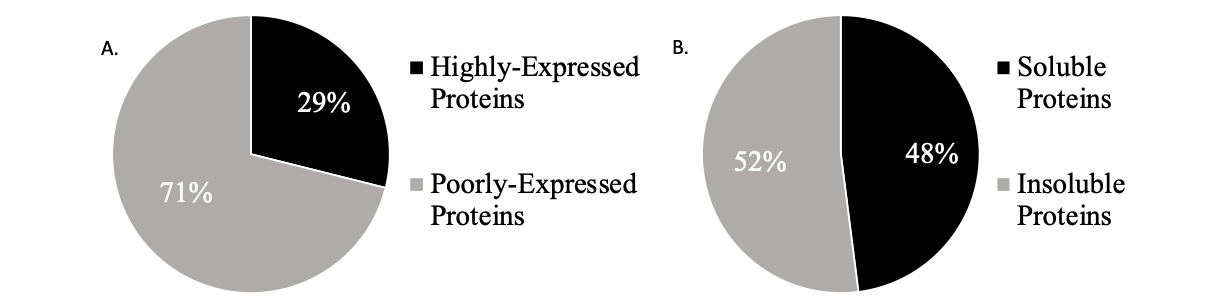
**

**Figure S1.** Expression levels and solubility of 173 fungal proteins expressed heterologously in *E. coli*. A. Percentage of different fungal proteins successfully expressed in *E. coli*. B. Percentage of different fungal proteins achieving soluble folding in *E. coli*.

 **Figure S2.** Comparative analysis of successfully expressed proteins with the overall protein population on different properties. **A.** Protein length, **B.** protein molecular weight, **C.** number of predicted N-linked glycosylation, **D.** number of predicted phosphorylation.

**
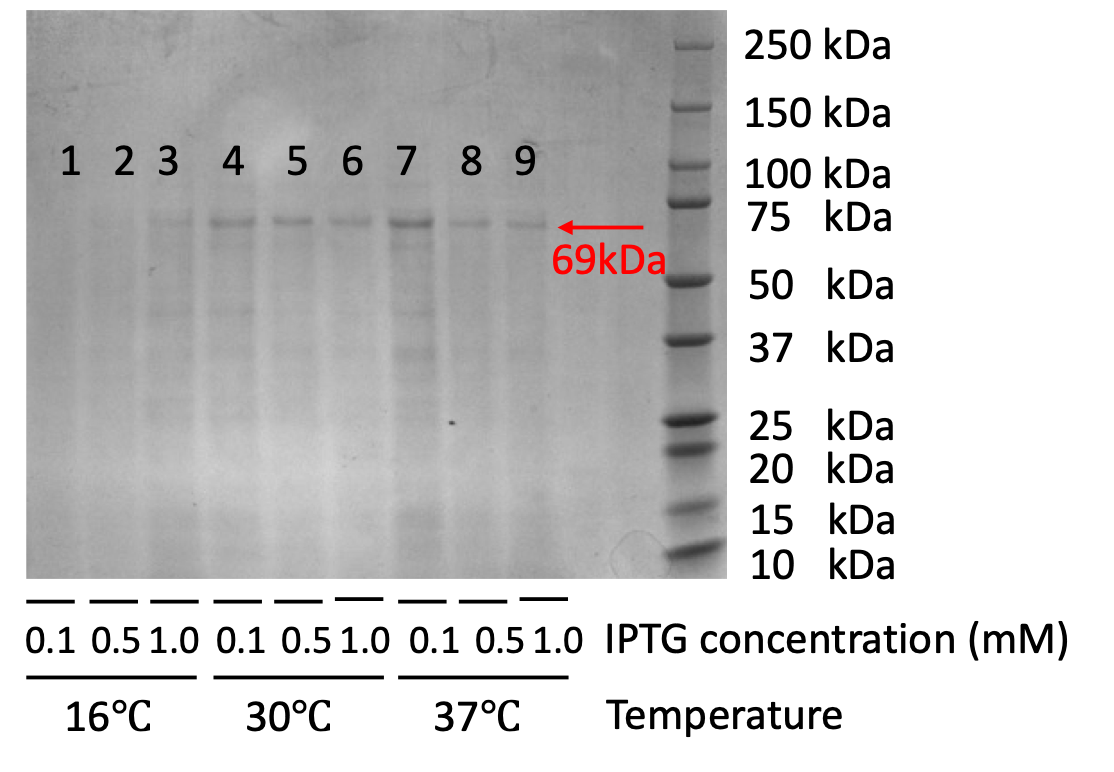
**

**Figure S3.** Optimization of parameters for fungal CAZyme IPTG-based inducible expression in *E. coli* heterologous system. Top numbers indicate each well where samples were loaded, and the protein ladder is shown in the rightmost lane with molecular weight next to the right band. The red arrow points to the band of the overexpressed celsome_012 proteins on an SDS-PAGE gel stained with Coomassie Blue. IPTG concentration used to induce expression is indicated, along with the temperature of expression.

**celsome_012:**

MGSSHHHHHHSSGLVPRGSHMASMYYTKVVQLLVTVGFVSAQWDWGNQWNWGNQWGNNGGNQMDPWGGNNGGNNNNNNGGYDDDTYVEREKVTPTQFFDENGAYFGPDCDSTNYSGAYYTGDYTSPFKTYLGKTDEEIQKKLDELWNHYFKGDNNSKVFYDQGNEGYILDVNNNDVRSEGMSYGMMIAVQTDHQKEFDKLWNWAKNHMWHKSGSWDGYFAWKRGSNGSGGDDNCAPDGEMYFMMSLLFAANRWNDKKYMDDAQYILKRMWDNSQHSLFNPAYNVITFQPQGNENNFSDPSYDLPAFVDLFSRWSTTNQNKWKAAASATRDHLYKSSNPQSGLFSDYNNFDGTPHSVSYNSNAQKYMYDAMRCAMNFGMDYYLFGTDAKRQTEMAKRIINHFEKDGYQHARFNWDGTGGSEQYTLGETGANAVATYALMGIAGYEDIIKKNLKMAWDGKPMTGQYRYYDGLVHYLSMLHLLGSFKIWKPAPSVQQKEISQTTVNGVTYKNGDVIDLFENCKLYKATINAGSSSQGTTPSNPSTGKCSSKITSQGYQCCSDNCEVIYTDADGNWGVENGEWCGCAKATSCIGAQGYPCCQSSTEVYFEDSDGKWSVENNDWCIIRN*

**Figure S4.** Proteomic analysis found celsome_012 protein was successfully produced via *E. coli* heterologous expression. Yellow highlighted amino acid residues are the peptides detected using mass spectrometry.

Tabel S1. List of predicted protein function based on protein structures

| **Gene ID** | **PDB ID** | **Chain** | **Annotation** | **EC** | **Length** | **Identity** | **Probability** |
| --- | --- | --- | --- | --- | --- | --- | --- |
| celsome_012 | 6trh | A | GH8 | 3.2.1.156 | 381 | 0.34 | 0.99 |
|  | 1e8q | A | CBM10 | na | 46 | 0.5 | 0.85 |
|  | 1e8q | A | CBM10 | na | 100 | 0.44 | 0.84 |
| celsome_039 | 5cxu | A | putatice esterase | 3.2.1.73 | 268 | 0.16 | 0.98 |
|  | 3pg0 | A | Threefoil | na | 140 | 0.33 | 0.99 |
|  | 1e8q | A | CBM10 | na | 100 | 0.44 | 0.84 |
| celsome_051 | 3wyd | B | peptidase S9, prolyl oligopeptidase, catalytic domain | 3.1.1.1 | 191 | 0.15 | 0.82 |
|  | 3pg0 | A | Threefoil | na | 140 | 0.31 | 1 |
| celsome_057 | 1vrx | B | GH5 | 3.2.1.4 | 358 | 0.51 | 1 |
|  | 1e8q | A | CBM10 | na | 46 | 0.53 | 0.95 |
| celsome_058 | 1ksc | A | GH9 | 3.2.1.4 | 433 | 0.42 | 0.94 |
|  | 1e8q | A | CBM10 | na | 46 | 0.4 | 0.95 |
|  | 1e8q | A | CBM10 | na | 46 | 0.43 | 0.95 |
|  | 1e8q | A | CBM10 | na | 46 | 0.55 | 0.84 |
| celsome_060 | 6idw | B | GH6 | 3.2.1 | 322 | 0.75 | 1 |
|  | 1e8q | A | CBM10 | na | 46 | 0.53 | 0.91 |
|  | 1e8q | A | CBM10 | na | 46 | 0.53 | 0.93 |
| celsome_061 | 3wug | A | GH10 | 3.2.1.8 | 312 | 0.31 | 1 |
|  | 3pg0 | A | Threefoil | na | 140 | 0.33 | 0.99 |
|  | 1e8q | A | CBM10 | na | 46 | 0.4 | 0.96 |
|  | 1e8q | A | CBM10 | na | 46 | 0.4 | 0.84 |
| celsome_069 | 3wug | A | GH10 | 3.2.1.8 | 312 | 0.31 | 1 |
|  | 3pg0 | A | Threefoil | na | 140 | 0.29 | 0.99 |
|  | 1e8q | A | CBM10 | na | 46 | 0.45 | 0.97 |
|  | 1e8q | A | CBM10 | na | 46 | 0.47 | 0.89 |
| celsome_071 | 6idw | B | GH6 | 3.2.1 | 322 | 0.52 | 1 |
|  | 6idw | B | GH6 | 3.2.1 | 322 | 0.52 | 0.98 |
|  | 1e8q | A | CBM10 | na | 46 | 0.45 | 0.89 |
|  | 1e8q | A | CBM10 | na | 46 | 0.51 | 0.84 |
| celsome_077 | 1ksc | A | GH9 | 3.2.1.4 | 433 | 0.41 | 0.96 |
|  | 1e8q | A | CBM10 | na | 46 | 0.21 | 0.85 |
|  | 1e8q | A | CBM10 | na | 46 | 0.43 | 0.91 |
| celsome_082 | 5xjo | D | Leucine Rich Repeat | 2.7.11.1 | 367 | 0.22 | 0.97 |
|  | 1e8q | A | CBM10 | na | 46 | 0.5 | 0.96 |
|  | 1e8q | A | CBM10 | na | 46 | 0.46 | 0.98 |
| celsome_089 | 2eac | A | GH65 | 3.2.1.63 | 881 | 0.26 | 1 |
|  | 3pg0 | A | Threefoil | na | 140 | 0.31 | 0.95 |
| celsome_120 | 1e8q | A | CBM10 | na | 46 | 0.47 | 0.97 |
|  | 1e8q | A | CBM10 | na | 46 | 0.5 | 0.97 |
| celsome_122 | 6qli | A | GH5 | 3.2.1.4 | 348 | 0.3 | 1 |
|  | 1e8q | A | CBM10 | na | 46 | 0.38 | 0.91 |
| celsome_148 | 5cxx | C | putative esterase | 3.1.1.73 | 272 | 0.68 | 1 |
| celsome_158 | 1vrx | B | GH5 | 3.2.1.4 | 368 | 0.51 | 1 |
|  | 1e8q | A | CBM10 | na | 46 | 0.55 | 0.93 |
|  | 1e8q | A | CBM10 | na | 46 | 0.47 | 0.91 |
|  | 1e8q | A | CBM10 | na | 46 | 0.53 | 0.92 |
| celsome_172 | 2apj | C | Carbohydrate esterase, Sialic acid-specidic acetylesterase | na | 240 | 0.18 | 0.97 |
|  | 4xuo | B | CBM4_9 | 3.2.1.8 | 156 | 0.25 | 0.99 |
|  | 1e8q | A | CBM10 | na | 46 | 0.54 | 0.97 |
|  | 1e8q | A | CBM10 | na | 46 | 0.62 | 0.92 |
